# Trends in Survival and Cure Indicators of Thin and Thick Cutaneous Malignant Melanoma in Italy

**DOI:** 10.1002/cam4.71486

**Published:** 2026-01-09

**Authors:** Silvia Mancini, Federica Toffolutti, Federica Zamagni, Lauro Bucchi, Emanuele Crocetti, Fabiola Giudici, Francesca Bella, Andrea Benedetto, Ettore Bidoli, Simona Carone, Giuliano Carrozzi, Giuseppe Cascone, Rossella Cavallo, Ilaria Cozzi, Fabio Falcini, Stefano Ferretti, Silvia Iacovacci, William Mantovani, Michael Mian, Maria Michiara, Maria Teresa Rocino, Tiziana Scuderi, Laura Ridolfi, Ignazio Stanganelli, Stefano Guzzinati, Luigino Dal Maso, AIRTUM Working Group, Vincenzo Amendola, Marta Bartiromo, Giuseppa Candela, Claudia Galluzzo, Maria Adalgisa Gentilini, Silvia Iacovacci, Chiara Lupi, Antonello Marras, Gabriele Morana, Antonino Musolino, Caterina Oriente, Caterina Palmonari, Diego Serraino, Rosario Tumino, Rosa Vattiato, Fabio Vittadello

**Affiliations:** ^1^ Romagna Cancer Registry, IRCCS Istituto Romagnolo per lo Studio dei Tumori (IRST) Dino Amadori Forlì Italy; ^2^ Cancer Epidemiology Unit Centro di Riferimento Oncologico di Aviano (CRO) IRCCS Aviano Italy; ^3^ Siracusa Cancer Registry, Provincial Health Authority of Siracusa Siracusa Italy; ^4^ Registro Tumori Integrato CT‐ME‐EN, Policlinico G. Rodolico San Marco di Catania Catania Italy; ^5^ Registro Tumori Puglia, sezione ASL Taranto, Struttura Complessa di Statistica ed Epidemiologia, Azienda sanitaria locale Taranto Taranto Italy; ^6^ Public Health Department Modena Cancer Registry, Local Health Authority Modena Italy; ^7^ Dipartimento di Prevenzione Asp Ragusa U.O.S.D. Registro Tumori di Ragusa e Caltanissetta Ragusa Italy; ^8^ Dipartimento di Prevenzione Registro Tumori Asl Salerno Salerno Italy; ^9^ Dipartimento di Epidemiologia SSR Lazio—ASL Roma 1 Roma Italy; ^10^ Cancer Prevention Unit Local Health Authority Forlì Italy; ^11^ Romagna Cancer Registry, Section of Ferrara, Local Health Authority, and University of Ferrara Ferrara Italy; ^12^ Latina Cancer Registry Lazio Italy; ^13^ Registro tumori di Trento—Servizio Epidemiologia Clinica e Valutativa, Azienda Provinciale per i Servizi Sanitari Trento Trento Italy; ^14^ Innovation, Research and Teaching Service (SABES‐ASDAA), Teaching Hospital of the Paracelsus Medical Private University (PMU) and College of Health Care‐Professions Claudiana Bolzano Italy; ^15^ Department of Medicine and Surgery, Medical Oncology Unit and Cancer Registry University of Parma, University Hospital of Parma Parma Italy; ^16^ Dipartimento di Prevenzione Asl Viterbo Viterbo Italy; ^17^ Dipartimento di Prevenzione ASP Trapani Registro Tumori di Trapani‐Agrigento Trapani Italy; ^18^ Advanced Cellular Therapy Unit and Rare Cancers, IRCCS Istituto Romagnolo per lo Studio dei Tumori (IRST) Dino Amadori Forlì Italy; ^19^ Skin Cancer Unit, IRCCS Istituto Romagnolo per lo Studio dei Tumori (IRST) Dino Amadori Forlì Italy; ^20^ Department of Dermatology University of Parma Parma Italy; ^21^ Epidemiological Department Azienda Zero Padova Italy

**Keywords:** cure, cure fraction, immune checkpoint inhibitors, melanoma, molecular targeted therapy, survival

## Abstract

**Background:**

In Italy, cure indicators of cutaneous malignant melanoma according to Breslow tumour thickness have never been assessed.

**Objectives:**

To evaluate the time trend in 1‐year net survival (NS), 5|1‐year conditional NS (CNS) and cure fraction (CF).

**Methods:**

Data from 10 cancer registries and 13,377 patients aged 15–74 years were used. Five|1‐year CNS was defined as the probability of surviving 5 years given that the patient has survived 1 year. CF was defined as the proportion of patients with the same life expectancy as the general population. One‐year NS and 5|1‐year CNS were contrasted between 2013–2017 and 2003–2007, and CF between 2015 and 2005.

**Results:**

For lesions up to 4 mm thick, 1‐year NS reached a level > 98.0%. In 2013–2017, 5|1‐year CNS was above 90% for men and women with lesions up to 2.0 mm thick and increased markedly for men with lesions > 2.0–4.0 mm thick (65.1% to 82.4%) and > 4.0 mm thick (57.6% to 69.4%). The CF of patients with a melanoma ≤ 1.0 mm thick was approximately 100% in both sexes, and nearly doubled from 2005 to 2015 (28% to 54%) for men aged 55–74 years with a melanoma > 4.0 mm thick.

**Conclusions:**

Patients with a melanoma ≤ 1 mm thick have the same life expectancy as the general population. The increase in the CF of men with thick lesions supports the hypothesis that novel therapies, approved in Italy since 2013, offer the possibility of cure.

## 1 Introduction

The worldwide increase in the incidence of cutaneous malignant melanoma (CMM) in the past decades [1, 2, 3] has been paralleled by a secondary prevention effort, with down‐staging of the disease [4, 5, 6] and a likely overdiagnosis phenomenon of unknown magnitude [7, 8, 9]. These trends have recently been followed by considerable therapeutic advances. Earlier detection and improved medical treatment have led to a marked increase in patient survival [6, 10, 11, 12]. All of these changes, coupled with population ageing, have resulted in an increasing number of individuals living after a diagnosis of CMM. In Europe, in 2020, their number was 779,000 [13]. In Italy, CMM is the cancer type with the most pronounced increase in prevalence, with an estimate of 270,000 in 2030 [14].

This scenario makes it increasingly necessary to improve our understanding of the long‐term prognosis of CMM. There are two main reasons for this. First, since the growing population of patients places a burden on healthcare services, follow‐up care should be tailored with precision. Unfortunately, there is currently no consensus regarding the type and total duration of post‐treatment clinical surveillance [15, 16]. And second, the novel drugs that have been introduced in the past decade, i.e., molecular targeted therapies and immune checkpoint inhibitors (ICI), are capable of achieving long‐term survival in a high proportion of patients with inoperable and metastatic disease, but it is still unclear whether this translates into cure [17], defined as the possibility to reach the same life expectancy as the general population [18]. For this reason, the long‐term survival of patients with CMM is more and more explored using the so‐called cure models [19, 20] in addition to net survival (NS) and conditional survival [6].

In Italy, a multicentre cancer registry‐based research project has documented the results of national policies for the control of CMM [2, 6, 21, 22]. According to a previously published study [6], the 5‐year NS in 2013–2017 improved versus 2003–2007, but particularly among men and in the two highest Breslow tumour thickness categories. Despite suffering from thicker lesions, men attained the same 5‐year NS as women (93.2% vs. 93.4%). The authors interpreted this apparent conundrum to be due to the advent of novel therapies for advanced CMM, first approved in Italy in 2013.

The objective of the present study was to complement the previous results on short‐term survival with estimates of cure indicators according to tumour thickness category.

## 2 Methods

### 2.1 Data Sources

Sixteen registries affiliated with the Italian Association of Cancer Registries (AIRTUM) accepted to participate in the study. Trained personnel extracted individual‐level records of people registered with primary CMM using the International Statistical Classification of Diseases and Related Health Problems, 10th revision, code C43 [23]. Even though not being included among the standard registration variables [24], tumour thickness has been regularly collected for years by several Italian cancer registries.

Six registries were excluded from the analysis because they did not fulfil the inclusion requirements of (i) having a proportion of cases with complete tumour thickness information ≥ 65% on a 3‐year basis (*n* = 4) and (ii) having complete follow‐up information until death or through 31 December 2021 (*n* = 2). There remained 10 registries available for inclusion (Table 1). Their number of years of registration varied between 10 and 21 with a median of 18.5. Nine of them took part in our previous study on 5‐year NS trends [6].

**TABLE 1:** Cancer registries participating in the study, period and number of years of registration, population covered and number of incident cases of cutaneous malignant melanoma (Italy, 1997–2017).

Cancer registry	Period of diagnosis	Number of years of registration	Population on 1 January 2017	Number of cases
Catania‐Messina‐ Enna	2003–2016	14	1,453,718	1935
Ferrara	2003–2014	12	258,475	493
Modena	1997–2017	21	519,218	1901
Parma	1997–2015	19	332,692	1263
Ragusa	1997–2017	21	446,048	771
Romagna	1997–2017	21	929,684	3982
Siracusa	1999–2016	18	307,547	498
Taranto	2006–2017	12	442,982	800
Trento	1997–2016	20	401,048	1268
Viterbo	2006–2015	10	242,202	466
All registries	1997–2017^a^	18.5^b^	5,333,614	13,377

*Note:* The study was restricted to cases aged 15–74 years.

^a^ Range.

^b^ Median number.

### 2.2 Study Population

Starting from 16,982 records, we excluded death‐certificate‐only cases, cases registered based on the autopsy report and cases with no follow‐up data, for a total of 46, and 3559 cases aged < 15 years or ≥ 75 years. The final dataset included 13,377 incident cases (1997–2017) from a resident population of 5,333,614 people (on 1 January 2017) aged 15–74 years. There were 6747 men (50.4%) and 6630 women (49.6%). Table S1 shows their distribution by patient age group, tumour site and clinico‐histologic subtype.

The ending date of follow‐up was 31 December 2021. The median duration was 10 years. For patients diagnosed in 2003–2007 and 2013–2017, undergoing selected subgroup analyses, the median follow‐up time was, respectively, 15 years and 6 years.

### 2.3 Definitions

One‐year NS was defined as the probability of surviving CMM 1 year after diagnosis, as calculated assuming that CMM was the only possible cause of death [25]. Expected survival was computed from the life tables made available by the Italian National Institute of Statistics for each registration area, stratified by patient age, sex and calendar year [26].

Five|1‐year conditional NS (CNS) was defined as the probability of surviving 5 years given that the patient has survived 1 year, that is, of surviving an additional 4 years. This measure was obtained from the NS at 1 + 4 years after diagnosis, with the time at risk being computed from 1 year after diagnosis.

The cure fraction (CF) represents the proportion of incident cases who experience the same life expectancy as their peers in the general population [18].

The time to cure (TTC) was defined as the number of years needed for patients to reach a 5‐year CNS > 95%, equivalent to a negligible excess mortality. In other words, TTC is the time after which the patients can be reasonably confident to have recovered from the disease, although they are still subject to the risk of death from other causes as the general population.

Further methodological details on definitions and validation of these indicators can be found elsewhere [18].

### 2.4 Data Analysis

One‐year NS and 5|1‐year CNS were contrasted between 2013–2017 and 2003–2007 in strata defined by sex, patient age (15–54 and 55–74 years) and tumour thickness.

For each tumour thickness category and sex, mixture cure models were applied to NS data separately by age group (15–54, 55–74 years). The period of diagnosis (1997–99, …, 2015–17) was used as a covariate. A Weibull distribution was obtained as a parametric function for the excess mortality of fatal cases, with independent parameters (shape and scale of Weibull distribution, period) for each tumour thickness category, sex and age group. All models were based on the assumption of linearity in the effect of the period of diagnosis. The assumption seems plausible within the period of diagnosis considered, since sudden changes in NS trends are rarely observed at the population level.

The CF was calculated using the mixture cure model as the NS at the attained age of 100 years, which was assumed to be the maximum age a person can live [18]. TTC was estimated as the number of years needed for the model‐based 5‐year CNS to exceed 95%. Figures S1 and S2 show the comparison between observed and model‐based NS and 5‐year CNS. CF and TTC were contrasted between 2013–2017 (centred in 2015) and 2003–2007 (centred in 2005), by sex, patient age and tumour thickness.

Tumour thickness was categorised according to the American Joint Committee on Cancer (AJCC) staging criteria, eighth edition [27], i.e., ≤ 0.8, > 0.8–1.0, > 1.0–2.0, > 2.0–4.0 and > 4.0 mm. Following a common approach in the relevant literature, the categories < 0.8 mm and 0.8–1.0 mm were both assumed to represent thin CMM and were pooled [5].

The statistical analysis was performed using SEERStat, Release 8.3.6 (National Cancer Institute, Bethesda, MD) and SAS (SAS Institute, Cary, NC).

## 3 Results

### 3.1 Patient Characteristics

Table 2 shows the distribution of the 13,377 eligible study cases by sex and tumour thickness. With a comparable proportion of CMMs of unknown thickness (10% vs. 8%), men had an approximately 10 percentage points smaller proportion of lesions ≤ 1.0 mm thick and an approximately 7 percentage points larger proportion of lesions > 2.0 mm thick.

**TABLE 2:** Number of incident cases of cutaneous malignant melanoma in Italy (1997–2017), by sex and Breslow tumour thickness category.

Tumour thickness (mm)	Men	Women	Total
	*n* (%)	*n* (%)	*n* (%)
≤ 1.0	3547 (52.6)	4110 (62.0)	7657 (57.2)
> 1.0–2.0	1030 (15.3)	998 (15.1)	2028 (15.2)
> 2.0–4.0	828 (12.3)	586 (8.8)	1414 (10.6)
> 4.0	640 (9.5)	390 (5.9)	1030 (7.7)
Unknown	702 (10.4)	546 (8.2)	1248 (9.3)
Any (total cases)	6747 (100.0)	6630 (100.0)	13,377 (100.0)

*Note:* The study was restricted to cases aged 15–74 years.

Table S2 shows the median age and the median tumour thickness in the time periods 2003–2007 and 2013–2017, by sex and tumour thickness. Among men, no clinically notable decrease in median thickness was observed in any tumour thickness category, and only negligible variations occurred among women.

### 3.2 One‐Year NS and 5|1‐Year CNS

In Table 3, 1‐year NS and 5|1‐year CNS in each tumour thickness category are contrasted between 2013–2017 and 2003–2007. For total lesions up to 4 mm thick, 1‐year NS was > 98.0% and stable for both sexes. Both for men and women with CMM > 4.0 mm thick, 1‐year NS increased moderately reaching, in this way, a level > 90.0%.

**TABLE 3:** Number, 1‐year net survival (%) and 5|1‐year conditional net survival (%) of incident cases of cutaneous malignant melanoma in Italy, by period of diagnosis (2003–2007, 2013–2017), sex and Breslow tumour thickness category.

	Survival measure	Tumour thickness (mm)	2003–2007		2013–2017	
			*n*	% (95% CI)	*n*	% (95% CI)
Men	1‐year NS^a^	≤ 1.0	679	100.0	1325	100.0
		> 1.0–2.0	253	99.8 (74.0; 100.0)	306	100.0
		> 2.0–4.0	192	98.6 (93.0; 99.7)	262	98.6 (94.8; 99.6)
		> 4.0	153	87.3 (80.5; 91.8)	208	92.7 (87.9; 95.7)
		Any (total cases)	1455	96.0 (94.7; 97.0)	2294	98.2 (97.4; 98.8)
	5|1‐year CNS^b^	≤ 1.0	676	99.5 (95.5; 100.0)	1314	99.9 (95.3; 100.0)
		> 1.0–2.0	251	89.4 (83.9; 93.2)	302	90.6 (85.7; 93.9)
		> 2.0–4.0	186	65.1 (57.2; 72.0)	255	82.4 (76.3; 87.1)
		> 4.0	132	57.6 (48.0; 66.0)	190	69.4 (61.6; 75.9)
		Any (total cases)	1383	86.6 (84.4; 88.6)	2225	92.9 (91.4; 94.2)
Women	1‐year NS^a^	≤ 1.0	936	100.0	1468	100.0
		> 1.0–2.0	266	98.4 (95.6; 99.4)	271	100.0
		> 2.0–4.0	141	98.4 (92.9; 99.7)	164	100.0
		> 4.0	83	87.3 (77.7; 92.9)	119	91.2 (84.2; 95.2)
		Any (total cases)	1558	97.9 (96.9; 98.5)	2185	98.7 (98.1; 99.1)
	5|1‐year CNS^b^	≤ 1.0	933	100.0	1462	99.5 (97.8; 99.9)
		> 1.0–2.0	261	91.4 (86.8; 94.4)	267	94.7 (90.7; 97.0)
		> 2.0–4.0	138	79.7 (71.3; 85.9)	162	87.1 (80.2; 91.7)
		> 4.0	72	67.3 (54.5; 77.2)	108	66.6 (56.0; 75.1)
		Any (total cases)	1518	93.8 (92.2; 95.0)	2139	95.8 (94.6; 96.7)

*Note:* The study was restricted to cases aged 15–74 years.

Abbreviations: CI, confidence interval; CNS, conditional net survival; NS, net survival.

^a^ Defined as the percent probability of surviving cutaneous malignant melanoma 1 year after diagnosis and calculated assuming that cutaneous malignant melanoma was the only possible cause of death.

^b^ Defined as the percent probability of surviving 5 years given that the patient has survived 1 year, that is, of surviving an additional 4 years. The estimate was adjusted for background mortality using the expected survival as obtained from the life tables provided the Italian National Institute of Statistics for each registration area, stratified by patient age (years), sex and calendar year.

Five|1‐year CNS was above 90% for men as well as women with CMM up to 2.0 mm thick. Men exhibited a marked improvement in the > 2.0–4.0 mm and > 4.0 mm thickness categories with women showing only a moderate increase in the former and none in the latter.

### 3.3 Cure Fraction

In 2005, the CF of CMM of any thickness was greater for women (age 15–54, 90% vs. 83%; age 55–74, 82% vs. 77%; overall, 86% vs. 79%). In 2015, all figures improved but the differences narrowed, particularly in the older age group (age 15–54, 94% vs. 89%; age 55–74, 85% vs. 84%; overall, 90% vs. 86%). Further details, including the CF of patients with missing tumour thickness information, can be found in Table S3.

**FIGURE 1:**
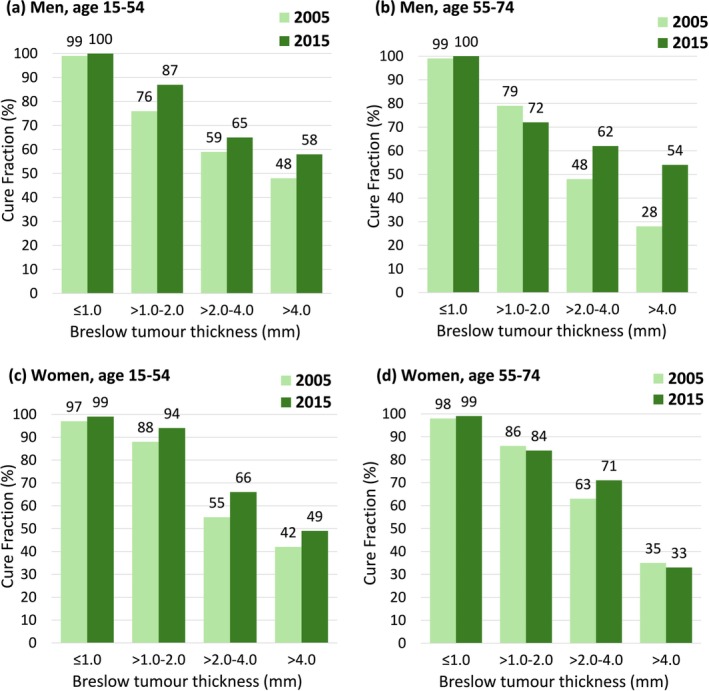
Cure fraction (%) of cases of cutaneous malignant melanoma in Italy, by sex, patient age group, Breslow tumour thickness category and year of diagnosis (2005, 2015). (a) men, age 15‐54; (b) men, age 55‐74; (c) women, age 15‐54; (d) women, age 55‐74.

In Figure 1, CF is contrasted between 2015 and 2005 by tumour thickness. The CF of patients with lesions ≤ 1.0 mm thick was approximately 100% in both sexes. The increase observed in 2015 was evident in the two categories of thickest lesions and especially among men. The greatest increase, nearly a doubling, occurred among men diagnosed with CMM > 4.0 mm thick at age 55–74 years (panel b). Thank to this, they reached a CF of over 50% which was greater than that of women.

### 3.4 Time to Cure

Between 2005 and 2015, the abovementioned increase in CF was accompanied by a decrease in TTC, which is shown in Table 4. In both years, TTC was < 1 year for patients with CMM ≤ 1.0 mm thick. For men diagnosed with CMM > 4.0 mm thick at age 55–74 years, TTC decreased from 12 to 10 years. Both in 2005 and 2015, men with CMMs thicker than 2.0 mm had a considerably shorter TTC than women, with a range of 7–12 years versus 10–20 years.

**TABLE 4:** Time to cure (years) of incident cases of cutaneous malignant melanoma in Italy, by year of diagnosis (2005, 2015), sex, patient age group and Breslow tumour thickness category.

Tumour thickness (mm)	2005				2015			
	Men		Women		Men		Women	
	15–54	55–74	15–54	55–74	15–54	55–74	15–54	55–74
≤ 1.0	1	1	1	1	1	1	1	1
> 1.0–2.0	9	10	1	5	5	11	1	6
> 2.0–4.0	10	11	> 15	11	9	10	14	10
> 4.0	7	12	> 15	15	7	10	> 15	15
Unknown	9	8	7	> 15	8	8	6	> 15
Any (total cases)	5	6	1	6	3	5	1	5

*Note:* The time to cure was defined as the number of years needed for patients to reach a 5‐year conditional net survival > 95%, equivalent to a negligible excess risk of death. When the estimated time to cure was < 1 year, it was set to 1 year. When time to cure was > 15 years represents patient groups with a significant excess risk of death that persists for more than 15 years.

**FIGURE 2:**
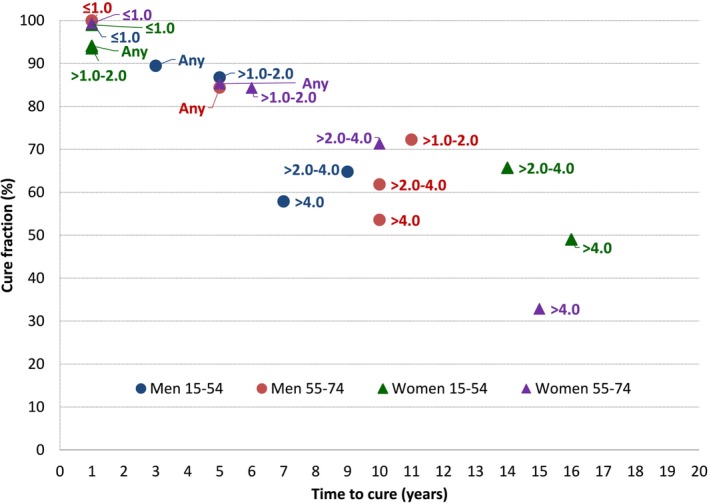
Scatterplot of cure fraction (%) and time to cure (years) of cases of cutaneous malignant melanoma in Italy (2015), by sex, patient age, and Breslow tumour thickness category. Any indicates any tumour thickness.

The inverse relationship between CF and TTC is confirmed by the scatterplot of the two indicators, by sex and tumour thickness category, in 2015 (Figure 2). Increasing CFs were associated with shorter TTCs.

## 4 Discussion

### 4.1 CMM ≤ 1.0 mm Thick

This study, which is unprecedented in Italy, adds two important insights into the long‐term prognosis of CMM. First, the large subgroup of patients diagnosed with a disease ≤ 1 mm thick exhibited a CF of 100% (men) and 99% (women), indicating that they had a negligible excess mortality compared with their peers in the general population. This finding is consistent with previous literature data [19, 28, 29] including our previous study on 5‐year NS trends [6], and confirms that patients with thin CMM in Italy have a life expectancy overlapping that observed in the general population. This may have an impact on survivorship care plans and favour a quick patients' return to the habitual life without encountering discrimination due to their past cancer diagnosis [30]. So far, only a minority of European Countries have adopted a legislation to regulate the ‘right to be forgotten’ of cancer patients [31], which is among the priorities of the Beating Cancer Plan [32].

### 4.2 CMM > 2.0–4.0 mm Thick and > 4.0 mm Thick

The changes occurring in the > 2.0–4.0 mm and > 4.0 mm tumour thickness categories were the second key finding of this study. In 2013–2017, 1‐year NS increased moderately both for men and women with CMM > 4.0 mm thick, reaching a level > 90%. With respect to 5|1‐year CNS, men exhibited a marked improvement in both thickness categories. More importantly, elderly men diagnosed with CMM > 4.0 mm thick in 2015 showed a doubling of the CF relative to 2005. This occurred in the absence of decreases in the median tumour thickness within this thickness category, which excludes the presence of a prognostic bias linked to an earlier detection. These observations add another piece of circumstantial evidence in support of the hypothesis that our previous data on survival from CCM, especially among men, were mostly explained by the introduction of molecular targeted therapies and ICI in 2013 [6]. Men suffer from a weaker resistance to disease progression [33, 34]. The immune system is impacted by the hormonal regulation and genetic differences, with women showing an upregulation of X‐chromosomal genes involved in the immune function [35, 36]. This makes further stimulation by ICI to be less effective and, consequently, men benefit from a larger overall survival increase when treated [37, 38]. It must be noted, however, that there are also negative studies that argue against an association between sex and the effectiveness of novel therapies for advanced melanoma [39, 40].

### 4.3 New Insights Into Novel Therapies

Our data allow a view into the targets and effects of novel therapies. As a rule, 1‐year NS is adversely affected by the prevalence of late‐stage, rapidly fatal cancers. By implication, an improvement is considered to reflect the down‐staging effect of an earlier detection [41]. However, this cannot be the case for this study, since the median tumour thickness within thickness categories did not change. We interpret the moderate improvement in 1‐year NS observed both in men and women with CMM > 4 mm thick as suggesting that the novel therapies can postpone some early deaths caused by otherwise rapidly fatal CMMs. In the past decade, a marked increase in 1‐year NS in U.S. patients with advanced and distant‐stage CMM has been attributed to the introduction of molecular targeted therapies and ICI since 2011 [42]. Similar changes in 1‐year relative survival were interpreted in the same manner in the Nordic Countries [43].

Five|1‐year CNS is negatively impacted by more delayed fatalities and, consequently, its improvements are interpreted to typically reflect improvements in systemic treatments. This is probably the explanation for the pronounced increase in 5|1‐year CNS of men with CMM > 2.0 mm thick, which occurred in the absence of any decrease in median tumour thickness. Conceivably, this gain too was the result of molecular targeted therapies and ICI. This would indicate that the novel therapies not only induce a response in advanced CMM, but might also impact on metastases that are occult at diagnosis and would clinically surface later. The use of ICI in the adjuvant setting in patients staged as AJCC IIB, IIC, IIIA, IIIB and IIIC has been approved [44].

### 4.4 Cure of and Mortality From CMM

Theoretically, the treatment of an advanced‐stage cancer can impact survival in two ways: by increasing the survival of non‐cured patients and by increasing the fraction of patients who are cured of their condition. This second effect has not been formally demonstrated [17]. The best circumstantial evidence so far is the dramatic fall in total age‐standardised mortality that has been observed in several western countries around mid‐2010s, that is, in temporal correlation with the introduction of molecular targeted therapies and ICI [45, 46, 47]. The present study adds further support to the hypothesis that these drugs are effective in obtaining the cure of advanced CMM, and not only in extending the survival of non‐cured patients. Our observation, however, needs to be corroborated with an Italian nationwide mortality study, considering that the persisting upward trend in incidence among middle‐aged and older adults [2], the associated high proportion of elderly patients and the reduced healthcare utilisation in southern Italy are expected to erode the impact of novel therapies on death rates. In this regard, it must be noted that the descriptive mortality statistics currently available in Italy do not show yet the dramatic decrease that, as stated above, is being currently observed in several high‐income countries [45, 46, 47].

### 4.5 Strengths and Limitations

The completeness and accuracy of cancer registries' data on survival in Italy represent an important strength of this study. The size of the study population and the duration of follow‐up both contributed to the reliability of results. The methods used for the estimates (in particular, of CF and TTC) are feasible and reproducible [48].

The study also suffers from two major limitations. First, it has a low‐resolution design. We had no access to information on treatment and prognostic factors except tumour thickness. For this reason, confounding of results was impossible to account for and the observed changes are of less straightforward interpretation. In particular, we had no access to the items of information needed to group cases according to the AJCC and SEER staging criteria and to identify unresectable diseases among patients with thick CMM. In fact, tumour thickness is only a proxy for tumour spread and, by implication, not all of those patients had indication for systemic treatment. This is equivalent to saying that the observed changes in the CF of patients –especially men– with thick lesions do probably underestimate the effect of novel therapies on this key cure indicator. The limited follow‐up time after their introduction is another reason for caution in evaluating the magnitude of changes in CF.

Second, the cure models too have potential limitations [49]. NS and cure indicators may have been affected by several biases, including lead time and length biases [50, 51]. We defined as ‘cured’ those patients who reached the same mortality rate as a comparable population free of cancer, with the assumption that cancer patients were exposed to the same risk factors as the general population. We omitted to present confidence intervals for CF and TTC and to perform sensitivity analyses for different TTCs [52, 53], in order to avoid overemphasised estimates of ‘precision’ by means of still largely debated variability measures [54]. Also, the threshold to define TTC (i.e., a low risk of recurrence/death) is arbitrary and is sensitive to the choice of the CNS threshold and to the use of different definitions and statistical models [48].

These considerations, however, have a limited impact on the estimate of TTC for CMM. As shown in Figures S1 and S2, the excess risk tended to decrease over time in all Breslow thickness strata. Also, the model‐based NS and 5‐year CNS fitted very well with the observed counterpart estimates in all thickness strata irrespective of patient age.

## 5 Conclusions

This study adds further evidence to the impact of early detection strategies on long‐term survival and cure of patients with CMM in Italy and suggests that molecular targeted therapies and ICI may increase the CF of patients with advanced disease in the real‐world setting.

## Author Contributions

**Silvia Mancini:** formal analysis, writing – review and editing. **Federica Toffolutti:** formal analysis, writing – review and editing. **Federica Zamagni:** writing – review and editing. **Lauro Bucchi:** conceptualisation, supervision, writing – original draft. **Emanuele Crocetti:** writing – review and editing. **Fabiola Giudici:** writing – review and editing. **Francesca Bella:** data curation, writing – review and editing. **Andrea Benedetto:** data curation, Writing – review and editing. **Ettore Bidoli:** data curation, writing – review and editing. **Simona Carone:** data curation, writing – review and editing. **Giuliano Carrozzi:** data curation, writing – review and editing. **Giuseppe Cascone:** data curation, writing – review and editing. **Rossella Cavallo:** data curation, writing – review and editing. **Ilaria Cozzi:** data curation, writing – review and editing. **Fabio Falcini:** data curation, writing – review and editing. **Stefano Ferretti:** data curation, writing – review and editing. **Silvia Iacovacci:** data curation, writing – review and editing. **William Mantovani:** data curation, Writing – review and editing. **Michael Mian:** data curation, writing – review and editing. **Maria Michiara:** data curation, writing – review and editing. **Maria Teresa Rocino:** data curation, writing – review and editing. **Tiziana Scuderi:** data curation, writing – review and editing. **Laura Ridolfi:** validation, writing – review and editing. **Ignazio Stanganelli:** validation, writing – review and editing. **Stefano Guzzinati:** conceptualisation, supervision, writing – review and editing. **Luigino Dal Maso:** conceptualisation, supervision, writing – original draft.

## Funding

This work was partly supported by the contribution of Ricerca Corrente by the Italian Ministry of Health, the AIRC IG (grant number 28893) and the Italian Melanoma Intergroup. The sponsors has no role in the study design; in the collection, analysis and interpretation of data; in the writing of the report; and in the decision to submit the article for publication.

## Ethics Statement

The study protocol was approved by the Ethics Committee at the Romagna Cancer Institute (ID: IRST100.37; date of approval: 22 July 2022; IRST identifier code: L1P1572).

## Consent

The authors have nothing to report.

## Conflicts of Interest

The authors declare no conflicts of interest.

## Supporting information


**Table S1.** Number of incident cases of cutaneous malignant melanoma in Italy (1997–2017), by patient age group, tumour site and clinico‐histologic subtype.
**Table S2.** Number, median patient age and median Breslow tumour thickness of incident cases of cutaneous malignant melanoma in Italy, by sex, period of diagnosis (2003–2007, 2013‐2017) and Breslow tumour thickness category.
**Table S3.** Cure fraction (%) of incident cases of cutaneous malignant melanoma in Italy, by sex, patient age group, Breslow tumour thickness category and year of diagnosis (2005, 2015).
**Figure S1.** Net survival and 5‐year conditional net survival of incident cases of cutaneous malignant melanoma in Italy and model‐based counterparts estimated until 18 years of follow, by Breslow tumour thickness. Patients (men and women pooled) aged 15–54 years, diagnosed in 2003–2005 and followed‐up until 2021.
**Figure S2.** Net survival and 5‐year conditional net survival of incident cases of cutaneous malignant melanoma in Italy and model‐based counterparts estimated until 18 years of follow, by Breslow tumour thickness. Patients (men and women pooled) aged 55–74 years, diagnosed in 2003–2005 and followed‐up until 2021.

## Data Availability

The data were used under license for this study and are available from the corresponding author with the permission of the Italian Association of Cancer Registries.

## The AIRTUM Working Group

Vincenzo Amendola (Modena Cancer Registry, Public Health Department, Local Health Authority, Modena); Marta Bartiromo (Registro Tumori Asl Salerno, Dipartimento di Prevenzione, Salerno); Giuseppa Candela (Registro Tumori di Trapani‐Agrigento, Dipartimento di Prevenzione ASP Trapani); Claudia Galluzzo (Registro Tumori Puglia, sezione ASL Taranto—Struttura Complessa di Statistica ed. Epidemiologia, Azienda sanitaria locale Taranto); Maria Adalgisa Gentilini (Registro tumori di Trento—Servizio Epidemiologia Clinica e Valutativa Azienda Provinciale per i Servizi Sanitari, Trento); Silvia Iacovacci (Latina Cancer Registry, Lazio); Chiara Lupi (Scuola di Specializzazione in Igiene e Medicina Preventiva dell'Università di Perugia e Registro Tumori dell'Umbria); Antonello Marras (Registro Tumori Integrato Ct‐ME‐En, Policlinico G. Rodolico San Marco di Catania); Gabriele Morana (Registro Tumori di Ragusa e Caltanissetta ASP Ragusa); Antonino Musolino (Department of Medicine and Surgery, University of Parma; Medical Oncology Unit and Cancer Registry, University Hospital of Parma, Parma); Caterina Oriente (Dipartimento di Prevenzione Asl Viterbo); Caterina Palmonari (Azienda USL Ferrara); Diego Serraino (Cancer Epidemiology Unit, Centro di Riferimento Oncologico di Aviano (CRO) IRCCS, Aviano); Rosario Tumino (Siracusa Cancer Registry, Provincial Health Authority of Siracusa, Siracusa); Rosa Vattiato (Romagna Cancer Registry, IRCCS Istituto Romagnolo per lo Studio dei Tumori (IRST) Dino Amadori, Meldola, Forlì); Fabio Vittadello (South Tyrol Cancer Registry, Bolzano).
