# Supplementary material for: Trends in Survival and Cure Indicators of Thin and Thick Cutaneous Malignant Melanoma in Italy
Source: Cancer Med. 2026 Jan 9;15(1):e71486. doi: 10.1002/cam4.71486 (PMC12788984; doi:10.1002/cam4.71486)
Supplement: Supplementary file 1 — Table S1. Number of incident cases of cutaneous malignant melanoma in Italy (1997–2017), by patient age group, tumour site and clinico‐histologic subtype. Table S2. Number, median patient age and median Breslow tumour thickness of incident cases of cutaneous malignant melanoma in Italy, by sex, period of diagnosis (2003–2007, 2013‐2017) and Breslow tumour thickness category. Table S3. Cure fraction (%) of incident cases of cutaneous malignant melanoma in Italy, by sex, patient age group, Breslow tumour thickness category and year of diagnosis (2005, 2015). Figure S1. Net survival and 5‐year conditional net survival of incident cases of cutaneous malignant melanoma in Italy and model‐based counterparts estimated until 18 years of follow, by Breslow tumour thickness. Patients (men and women pooled) aged 15–54 years, diagnosed in 2003–2005 and followed‐up until 2021. Figure S2. Net survival and 5‐year conditional net survival of incident cases of cutaneous malignant melanoma in Italy and model‐based counterparts estimated until 18 years of follow, by Breslow tumour thickness. Patients (men and women pooled) aged 55–74 years, diagnosed in 2003–2005 and followed‐up until 2021. [file CAM4-15-e71486-s001.doc]

**Trends in survival and cure indicators of thin and thick cutaneous malignant melanoma in Italy**

Silvia Mancini^1^, Federica Toffolutti^2^, Federica Zamagni^1^, Lauro Bucchi^1^, Emanuele Crocetti^1^, Fabiola Giudici^2^, Francesca Bella^3^, Andrea Benedetto^4^, Ettore Bidoli^2^, Simona Carone^5^, Giuliano Carrozzi^6^, Giuseppe Cascone^7^, Rossella Cavallo^8^, Ilaria Cozzi^9^, Fabio Falcini^1, 10^, Stefano Ferretti^11^, Silvia Iacovacci^12^, William Mantovani^13^, Michael Mian^14^, Maria Michiara^15^, Maria Teresa Rocino^16^, Tiziana Scuderi^17^, Laura Ridolfi^18^, Ignazio Stanganelli^19,20^, Stefano Guzzinati^21^, Luigino Dal Maso^2^, AIRTUM Working Group†

^1^Romagna Cancer Registry, IRCCS Istituto Romagnolo per lo Studio dei Tumori (IRST) Dino Amadori, Meldola, Forlì, Italy.

^2^Cancer Epidemiology Unit, Centro di Riferimento Oncologico di Aviano (CRO) IRCCS, Aviano, Italy

^3^Siracusa Cancer Registry, Provincial Health Authority of Siracusa, Siracusa, Italy.

^4^Registro Tumori Integrato CT-ME-EN, Policlinico G. Rodolico San Marco di Catania, Italy.

^5^Registro Tumori Puglia, sezione ASL Taranto - Struttura Complessa di Statistica ed Epidemiologia, Azienda sanitaria locale Taranto, Italy.

^6^Modena Cancer Registry, Public Health Department, Local Health Authority, Modena, Italy.

^7^U.O.S.D. Registro Tumori di Ragusa e Caltanissetta Dipartimento di prevenzione Asp Ragusa, Italy.

^8^Registro Tumori Asl Salerno, Dipartimento di Prevenzione, Italy

^9^Dipartimento di Epidemiologia SSR Lazio - ASL Roma 1, Roma, Italy.

^10^Cancer Prevention Unit, Local Health Authority, Forlì, Italy.

^11^Romagna Cancer Registry, Section of Ferrara, Local Health Authority, and University of Ferrara, Ferrara, Italy.

^12^Latina Cancer Registry, Lazio, Italy.

^13^Registro tumori di Trento - Servizio Epidemiologia Clinica e Valutativa Azienda Provinciale per i Servizi Sanitari Trento, Italy.

^14^Innovation, Research and Teaching Service (SABES-ASDAA), Teaching Hospital of the Paracelsus Medical Private University (PMU) and College of Health Care-Professions Claudiana, Bolzano, Italy.

^15^Department of Medicine and Surgery, University of Parma; Medical Oncology Unit and Cancer Registry, University Hospital of Parma, Parma, Italy

^16^Dipartimento di Prevenzione Asl Viterbo, Italy.

^17^Registro Tumori di Trapani-Agrigento, Dipartimento di Prevenzione ASP Trapani, Italy.

^18^Advanced Cellular Therapy Unit and Rare Cancers, IRCCS Istituto Romagnolo per lo Studio dei Tumori (IRST) Dino Amadori, Meldola, Forlì, Italy.

^19^Skin Cancer Unit, IRCCS Istituto Romagnolo per lo Studio dei Tumori (IRST) Dino Amadori, Meldola, Forlì, Italy.

^20^Department of Dermatology, University of Parma, Parma, Italy.

^21^Epidemiological Department, Azienda Zero, Padova, Italy,

**Correspondence**

Lauro Bucchi, Romagna Cancer Registry, IRCCS Istituto Romagnolo per lo Studio dei Tumori (IRST) Dino Amadori, Meldola, Forlì, Italy.

Email: [lauro.bucchi@irst.emr.it](mailto:lauro.bucchi@irst.emr.it)

**SUPPORTING INFORMATION**

**Table S1 p. 2**

**Table S2 p. 2**

**Table S3 p. 3**

**Figure S1 p. 4-5**

**Figure S2 p. 6-7**

TABLE S1. Number of incident cases of cutaneous malignant melanoma in Italy (1997-2017), by patient age group, tumour site and clinico-histologic subtype.

|  | Men |  | Women |  | Total |
| --- | --- | --- | --- | --- | --- |
|  | *n* (%) |  | *n* (%) |  | *n* (%) |
| Age at diagnosis (years) |  |  |  |  |  |
| 15-54 | 3134 (46.5) |  | 4015 (60.6) |  | 7149 (53.4) |
| 55-64 | 1689 (25.0) |  | 1316 (19.8) |  | 3005 (22.5) |
| 65-74 | 1924 (28.5) |  | 1299 (19.6) |  | 3223 (24.1) |
| Tumour site |  |  |  |  |  |
| Head and neck | 702 (10.4) |  | 415 (6.3) |  | 1117 (8.4) |
| Trunk | 3437 (50.9) |  | 2196 (33.1) |  | 5633 (42.1) |
| Upper limb | 1170 (17.3) |  | 1146 (17.3) |  | 2316 (17.3) |
| Lower limb | 1008 (14.9) |  | 2474 (37.3) |  | 3482 (26.0) |
| Others | 430 (6.4) |  | 399 (6.0) |  | 829 (6.2) |
| Clinico-histologic subtype |  |  |  |  |  |
| Superficial spreading melanoma | 2895 (42.9) |  | 3188 (48.1) |  | 6083 (45.5) |
| Nodular melanoma | 733 (10.9) |  | 464 (7.0) |  | 1197 (8.9) |
| Lentigo maligna melanoma | 102 (1.5) |  | 95 (1.4) |  | 197 (1.5) |
| Others | 2025 (30.0) |  | 2028 (30.6) |  | 4053 (30.3) |
| NS | 992 (14.7) |  | 855 (12.9) |  | 1847 (13.8) |
| Total | 6747 (100.0) |  | 6630 (100.0) |  | 13,377 (100.0) |

Abbreviation: NS, not specified.

TABLE S2. Number, median patient age and median Breslow tumour thickness of incident cases of cutaneous malignant melanoma in Italy, by sex, period of diagnosis (2003-2007, 2013-2017) and Breslow tumour thickness category.

|  | Tumour thickness (mm) | Men | | |  | Women | | |
| --- | --- | --- | --- | --- | --- | --- | --- | --- |
|  |  | *n* | Median patient age (years) | Median tumour thickness (mm) |  | *n* | Median patient age (years) | Median tumour thickness (mm) |
| 2003-2007 | ≤1.0  >1.0-2.0  >2.0-4.0  >4.0  Unknown  Any (total cases) | 679  253  192  153  178  1455 | 53  55  62  61  58  56 | 0.52  1.40  3.00  6.00  NC  0.90 |  | 936  266  141  83  132  1558 | 47  48  58  60  52  49 | 0.52  1.40  3.00  7.10  NC  0.74 |
| 2013-2017 | ≤1.0  >1.0-2.0  >2.0-4.0  >4.0  Unknown  Any (total cases) | 1325  306  262  208  194  2295 | 55^a^  56  60  63  56  57 | 0.50^a^  1.35  3.00  7.00  NC  0.73^c^ |  | 1468  271  164  119  164  2186 | 49^a^  51  53  58  50  50 | 0.49^b^  1.35  2.80  7.00  NC  0.62^c^ |

Abbreviation: NC, not computable.

*Note:* The study was restricted to cases aged 15-74 years. For both sexes, the distribution by patient age and tumour thickness, as continuous variables, was compared between 2003-2007 and 2013-2017 in each single tumor thickness category using the Pearson chi-squared test of the equality of medians. Non-significant distribution differences are not indicated.

^a^*p*<0.05.

^b^*p*<0.01.

^c^*p*<0.001.

TABLE S3. Cure fraction (%) of incident cases of cutaneous malignant melanoma in Italy, by sex, patient age group, Breslow tumour thickness category and year of diagnosis (2005, 2015).

|  | Men | | |  | Women | | |
| --- | --- | --- | --- | --- | --- | --- | --- |
|  | All ages  (15-74 years) | 15-54  years | 55-74  years |  | All ages  (15-74 years) | 15-54  years | 55-74  years |
|  | Cure fraction centred in 2005 | | | | | | |
| ≤1.0 | 99 | 99 | 99 |  | 98 | 97 | 98 |
| >1.0-2.0 | 78 | 76 | 79 |  | 87 | 88 | 86 |
| >2.0-4.0 | 51 | 59 | 48 |  | 60 | 55 | 63 |
| >4.0 | 32 | 48 | 28 |  | 36 | 42 | 35 |
| Unknown | 50 | 59 | 46 |  | 49 | 70 | 35 |
| Any (all cases) | 79 | 83 | 77 |  | 86 | 90 | 82 |
|  | Cure fraction centred in 2015 | | | | | | |
| ≤1.0 | 100 | 100 | 100 |  | 99 | 99 | 99 |
| >1.0-2.0 | 77 | 87 | 72 |  | 88 | 94 | 84 |
| >2.0-4.0 | 63 | 65 | 62 |  | 69 | 66 | 71 |
| >4.0 | 54 | 58 | 54 |  | 36 | 49 | 33 |
| Unknown | 55 | 64 | 51 |  | 49 | 74 | 28 |
| Any (all cases) | 86 | 89 | 84 |  | 90 | 94 | 85 |

**≤1 mm**

**>1.0-2.0 mm**

FIGURE S1. Net survival and 5-year conditional net survival of incident cases of cutaneous malignant melanoma in Italy and model-based counterparts estimated until 18 years of follow-up, by Breslow tumour thickness. Patients (men and women pooled) aged 15-54 years, diagnosed in 2003-2005 and followed-up until 2021. Abbreviations: NS, net survival; CNS, conditional net survival. *Note:* At the bottom of each panel, the number of patients alive at 0, 5, 10, and 15 years since diagnosis is displayed.

**>2.0-4.0 mm**

**>4.0 mm**

FIGURE S1. Continued.

**≤1 mm**

**>1.0-2.0 mm**

FIGURE S2. Net survival and 5-year conditional net survival of incident cases of cutaneous malignant melanoma in Italy and model-based counterparts estimated until 18 years of follow-up, by Breslow tumour thickness. Patients (men and women pooled) aged 55-74 years, diagnosed in 2003-2005 and followed-up until 2021. Abbreviations: NS, net survival; CNS, conditional net survival. *Note:* At the bottom of each panel, the number of patients alive at 0, 5, 10, and 15 years since diagnosis is displayed.

**>2.0-4.0 mm**

**>4.0 mm**

FIGURE S2. Continued.
